# Stereotactic Body Therapy for Urologic Cancers—What the Urologist Needs to Know

**DOI:** 10.3390/life14121683

**Published:** 2024-12-19

**Authors:** Jasamine Coles-Black, Adib Rahman, Shankar Siva, Joseph Ischia, Marlon Perera, Damien Bolton, Nathan Lawrentschuk

**Affiliations:** 1 Department of Surgery, Austin Health, The University of Melbourne, Parkville, VIC 3010, Australia; ischiajj@hotmail.com (J.I.); marlonlperera@gmail.com (M.P.); damienmbolton@gmail.com (D.B.); 2 Department of Surgery, Redcliffe Hospital, Redcliffe, QLD 4020, Australia; adib.rahman3@gmail.com; 3 Department of Radiation Oncology, Peter MacCallum Cancer Centre, The University of Melbourne, Parkville, VIC 3052, Australia; shankar.siva@petermac.org; 4 Department of Surgery, Peter MacCallum Cancer Centre, The University of Melbourne, Parkville, VIC 3052, Australia; lawrentschuk@gmail.com; 5 Department of Surgery, The Royal Melbourne Hospital, The University of Melbourne, Parkville, VIC 3052, Australia

**Keywords:** SABR, SBRT, radiotherapy, urology, oncology, uro-oncology, urologic cancers, prostate cancer, renal cell carcinoma

## Abstract

Background: stereotactic ablative body radiotherapy (SABR) is a disruptive radiation therapy technique which is increasingly used for the treatment of urologic cancers. The aim of this narrative review is to provide an overview on the current landscape of SABR in urologic cancers and highlight advancements on the horizon. Methods: a narrative review of the contemporary role of SABR in urologic cancers is conducted. Results: in localised prostate cancer, SABR boasts excellent tumour control and biochemical control, with acceptable GU and GI toxicity. Its comparison to laparoscopic radical prostatectomy is currently ongoing. SABR appears to be practical for metastasis-directed therapy in metastatic prostate cancer, with good local control and a low toxicity profile, either alone or in combination with ADT. In localised RCC, SABR offers adequate local control with a modest impact on renal function in patients unfit for surgical management. Its role in metastatic RCC is much more established, where it has been shown to be superior to conventional radiotherapy. Emerging evidence suggests that SABR has a role in delaying systemic therapy whilst maintaining QOL and overall survival. Intriguingly, in metastatic prostate cancer and metastatic RCC, SABR results in a cytoreductive and immunomodulatory ‘abscopal effect’, a focus of current investigations. Conclusions: SABR has emerged as a safe, effective, and feasible treatment for urologic cancers. Urologists should be aware of its increasing use in localised prostate cancer and metastatic RCC, with good oncological outcomes combined with acceptable toxicity. In addition, SABR holds promise for both metastatic prostate cancer and localised RCC treatment in terms of toxicity and oncological outcomes.

## 1. Introduction

Radiation therapy is a widely accepted treatment for localised and advanced prostate cancer, with the use of radiation for localised prostate cancer first reported in 1909 [1]. In contrast to this, established historical principles regarding the radioresistance of renal cell carcinoma (RCC) have been recently overturned by new advances in radiation planning and delivery, extending its application to localised and metastatic RCC in this rapidly evolving field of study.

Stereotactic ablative body Rradiotherapy (SABR) is a disruptive radiation therapy technique which is increasingly used for the treatment of urologic cancers and is now a standard treatment option for many tumours of the lung, liver, brain, spine, and pancreas [2]. This narrative review seeks to provide an updated overview of SABR’s role in managing urologic cancers and highlight anticipated advancements.

SABR, also known as Stereotactic Body Radiotherapy (SBRT), is a form of external beam radiation therapy (ERBT) that delivers higher doses of radiation per fraction of therapy. This allows high doses of radiation (namely 7.25–10 GY per fraction) to be administered to patients in a very short timeframe, typically during five sessions or less. The American Society of Radiation Oncology (ASTRO) defines SABR as “an ERBT method used to precisely deliver a high dose of radiation to an extracranial target within the body, using either a single dose or a small number of fractions. Specialised treatment planning results in high target dose and steep dose gradients beyond the target” [3].

Conventional ERBT is delivered in small doses over many fractions to maintain a therapeutic window, balancing toxicity to the surrounding normal tissue with tumour control. For example, in localised prostate cancer, conventional ERBT is non-invasive with a widely accepted side-effect profile. Yet, treatment with 39–44 fractions over 8–9 weeks increases health care costs and creates logistical challenges for patients, compromising their quality of life (QOL) [4]. Additionally, SABR is performed in an awake patient without the need for anaesthesia or sedation. Decreasing the number of fractions, referred to as a hypofractionated or even ultrahypofractionated treatment, reduces the burden of time spent receiving treatment on the patients [5]. When ultrahypofractionation is employed in five or fewer regimens via novel delivery systems, it is referred to as SABR [4].

## 2. SABR in Localised Prostate Cancer

There have been several advances in the delivery of radiation to localised prostate cancer, such as increasing the dose per fraction to ultrahypofractionated regimens in SABR (≥5 Gy per fraction), allowing radiation oncologists to deliver more precise pre-procedural planning and treatment.

Firstly, the uniquely low alpha–beta ratio of prostatic tissue at 1.5 (0.9–2.2) Gy allows the radiobiology of prostate cancer cells to be exploited by the advent of SABR [6]. The alpha–beta ratio describes cell survival as a function of radiation dose per fraction, meaning that prostate cells are more likely to survive small doses of radiation but are synchronously more likely to be destroyed with a larger fraction size [7].

Secondly, improvements in imaging and radiation delivery have enabled the progress of SABR. When administering radiation, consistency in patient positioning, target visualisation, and target motion results in a planned margin of error surrounding the region of interest to ensure adequate dosing to the prostate, while accounting for prostatic mobility and minor variations in positioning. The prostate has been shown to exhibit significant movement during and between treatment sessions [8], increasing the risk of off-target radiation and greater toxicity. Geometric uncertainty in prostate positioning arises from multiple factors, including volume contouring errors, setup errors, and target position variations, all of which contribute to treatment inaccuracies. While the bladder and rectal physiology, as well as the breathing motion, can affect target position, rectal filling has been found to exert the most significant impact on target position [8]. This has been addressed through contemporary image-guided radiotherapy (IGRT) and the use of radiolucent fiducial markers placed within the targeted prostatic tissue to direct radiation during and between treatment sessions [4], narrowing the margin of error.

Narrower margins minimise the dose received by intimately related structures such as the bladder and rectum, with the achievement of narrower margins further improved by the introduction of intensity-modulated radiotherapy (IMRT). This technique conforms the geometry of the radiation beam to allow the intended target to receive a high dose while further minimising the dose received by the surrounding structures [9].

IGRT and IMRT are two advances largely responsible for the mainstream feasibility of SABR as a technique. A systematic review and meta-analysis performed by Jackson et al. pooled 6116 men with localised prostate cancer across 36 prospective studies with a median follow-up of 39 months, reporting on two key findings [2].

Firstly, SABR resulted in excellent tumour control. Fourteen studies (*n* = 2343) reported a 95.3% 5-year biochemical recurrence-free survival (95% CI, 91.3–97.5%, *p* < 0.001), with an increased dose significantly associated with improved biochemical recurrence-free survival (*p* = 0.018). This could be further categorised by prostate cancer risk classification: 95.0–100% for low-risk disease, 90.7–100% for favourable intermediate-risk disease, and 81.0–93.1% for unfavourable intermediate-risk disease.

Secondly, increasing the SABR dose was associated with improved biochemical control (*p* = 0.018), but it resulted in worse late grade ≥ 3 genitourinary (GU) toxicity (*p* = 0.014). Combined acute GU and gastrointestinal (GI) toxicity of grade ≥ 3 was very rare at ≤1%. Late grade ≥ 3 GU toxicity was 2% (95% CI, 1.4–2.8%, *p* = 0.083), and late grade 3 GI toxicity was 1.1% (95% CI, 0.6–2.0%, *p* < 0.001). GU and GI toxicities per common terminology criteria for adverse events (CTCAE) are categorised into five grades (0–5): grade 0 indicates no adverse events, grade 1 indicates mild symptoms not requiring intervention, grade 2 indicates moderate symptoms necessitating minor, local, or non-invasive intervention, grade 4 indicates life-threatening or disabling events requiring urgent intervention, and grade 5 indicates death related to the adverse event.

A total of 30 studies (*n* = 5127) were analysed, revealing a significant association with increasing doses and late grade ≥ 3 GU toxicity; nevertheless, there was no association between dose and late grade ≥ 3 GI toxicity (*p* = 0.56). With respect to patient-reported QOL, the international prostate symptom score (IPSS) and the expanded prostate cancer index composite short form (EPIC-26) scores were collated. Due to the heterogenous reporting of IPSS scores, only EPIC-26 data were extracted for urinary, bowel, and sexual domains, revealing that urinary and bowel scores returned to baseline 2 years post-SABR (*p* = 0.9 and *p* = 0.09, respectively) and remained similar 5 years after SABR (*p* = 0.50 and *p* = 0.80, respectively). Sexual domain scores gradually decreased over time, with statistical significance achieved 3 years following SABR (*p* = 0.01).

The study, therefore, concluded that SABR should be considered a standard treatment option for localised prostate cancer, even suggesting further trials to assess its potential superiority when compared to other techniques [2].

### 2.1. Comparison with ERBT

SABR may facilitate additional pathways for prostate cancer cell death not delivered by ERBT, such as increasing the prostate cancer expression of inflammatory mediators, cytokines, and death receptors. Additionally, indirect tumour death via ceramide-mediated apoptosis of the surrounding vascular endothelium has been suggested to play a role [10]. Regarding safety and efficacy, most studies to date constitute phase I-II trials and single-centre studies, demonstrating similar toxicity and noninferior oncological control compared to conventional ERBT [11].

However, two recent phase III trials have demonstrated equivalent toxicity, with oncological outcomes yet to be reported. The PACE-B trial was a phase III open-label, multicohort, randomised controlled trial enrolling 874 men with low-to-intermediate-risk prostate cancer from the UK, Ireland, and Canada [12]. Men were stratified by centre and risk group into an ERBT (control) group (441 men receiving 78 Gy in 39 fractions over 7–8 weeks/62 Gy in 20 fractions over 4 weeks) or into a SABR group (443 men receiving 36 Gy in five fractions over 1–2 weeks). Acute toxicity was found to be an important predictor of both late GU and GI toxicity after localised prostate radiotherapy following conventional ERBT and SABR, with the investigators proposing that optimisation of these symptoms prior to treatment and a more aggressive early intervention could prevent the development of late toxicity [13]. The study suggested that substantially shortening the treatment courses by greatly increasing the radiation doses did not increase either GU or GI acute toxicity. This finding contrasts with that of the earlier HYPO-RT-PC, an earlier randomised, controlled, non-inferiority phase III trial which used older conventional radiotherapy techniques delivered over a shorter treatment period.

HYPO-RT-PC compared failure-free survival in 1200 men with intermediate- and high-risk prostate cancer receiving ultrahypofractionated RT (42.7 Gy in seven fractions over 2.5 weeks) or conventional ERBT (79 Gy in 38 fractions) [14]. Patients reported significantly worse acute toxicity via the prostate cancer symptom scale (PCSS) in the SABR group and remained in a significantly worse state 3 months after the treatment. Despite this, in a subsequent 6-year follow-up study, there was no difference in the long-term patient-reported QOL [15], further supporting the utility of ultrahypofractionation for intermediate-to-high-risk prostate cancer.

While both trials endorsed the radiobiological premise of ultrahypofractionation, the discrepancy between the acute toxicity of SABR in HYPO-RT-PC and the findings from the more contemporary PACE-B may firstly be due to the use of three-dimensional conformal radiotherapy in HYPO-RT-PC, an older planning technique compared to IMRT, and to the wider margins compared to those occurring with modern treatment planning, subjecting the surrounding structures to higher absolute values of radiation when compared to PACE-B [14].

### 2.2. Large Prostates

Patients with large prostate volumes have been shown to have higher rates of GU and GI toxicity following ERBT. Given its nascency, the toxicity of SABR has been less well studied in this patient population. Janowski et al. followed up 57 men (23 low-, 25 intermediate-, and 9 high-risk) with a median age of 69 years who received SABR with a median follow-up of 2.9 years. The median prostate size was 62.9 cm^3^ (range 50–138.7 cm^3^) and one third of the patients received ADT. SABR for clinically localised prostate cancer was well tolerated in this cohort; the 2-year incidences of GU and GI toxicity were 49.1% and 1.8%, with two patients experiencing grade 3 GU toxicity [16]. A recent review published by Martin et al. highlights that patients with a larger prostate should be considered for formal urodynamics and mini-TURP when associated with obstructive LUTS [17].

### 2.3. Comparison with Brachytherapy

Like SABR, high-dose-rate brachytherapy (HDR-BT) is another technique for the delivery of ultrahypofractionation. A recent study by Yoshioka et al. compared the doses delivered to the surrounding tissues in HDR-BT (*n* = 20), robotic (*n* = 40), and non-robotic SABR (*n* = 40) between 2018 and 2022 [18]. Consistent with previous evidence, intraprostatic doses were consistently higher, with HDR-BT comparable to SABR [19,20,21]. HDR-BT resulted in statistically significantly lower doses to the bladder and rectum compared to robotic and non-robotic SABR. Conversely, the dose to the urethra was slightly but statistically significantly higher following the use of HDR-BT compared to both SABR techniques. Robotic SABR, in particular, resulted in a significantly lower urethral dose compared to HDR-BT.

Regarding patient QOL, trials on combination brachytherapy for intermediate- or high-risk diseases have reported grade 3–4 GU and GI toxicity at 10–30% on follow-up [22,23,24]. SABR compares very favourably with this, with a recent systematic review and meta-analysis of SABR for localised prostate cancer treatment (*n* = 6116) reporting GU and GI grade 3–4 toxicity at <2.5% [2]. As with ERBT, the long-term oncological outcomes of brachytherapy compared to SABR are a focus of future investigations, but existing data on hypofractionated regimens appear promising.

### 2.4. Comparison with Radical Prostatectomy

Katz et al. used the EPIC scoring system to retrospectively assess the impact of SABR compared to open radical retropubic prostatectomy on GU, GI, sexual function, and QOL in prostate cancer patients [25]. Between 2006 and 2008, 216 SABR patients received a total dose of 35–36.2 Gy in five daily fractions. In the surgery group, an open radical retropubic approach was used between 2003 and 2005, with nerve-sparing techniques implemented in 28% of the patients.

One-to-six months following treatment, radical prostatectomy patients reported larger declines in GU and sexual QOL compared to the SABR group, with a larger decline in GI QOL following SABR compared to surgery. Long-term (36 months) urinary and sexual QOL remained clinically and significantly reduced in surgery patients but not in the SABR group, with no longer term data on QOL beyond 36 months.

Notably, there were differences between the two intervention groups. The SABR patients were older, with lower baseline PSA values. The SABR group had a significantly greater proportion of low-risk diseases compared to the surgery group (156,72.2% vs. 52, 42.3%) and a significantly lower proportion of intermediate-risk diseases (56, 25.9% vs. 67, 54.5%). In addition, baseline and continuing sildenafil use was greater in the older SABR patients, with fewer younger open radical prostatectomy patients using pharmacologic aids.

These limitations necessitate equivalent comparisons among more contemporary techniques, given the modern improvements in both approaches. SABR has seen increasing mainstream use over the last two decades [2]. In this timeframe, robot-assisted radical prostatectomy has become the preferred minimally invasive approach, where available [26].

The PACE-A trial is a phase III multicentre, randomised study comparing efficacy, toxicity, and QOL outcomes in men with organ-confined low- and intermediate-risk prostate cancer treated with SABR (63 men receiving 36.25 Gy in five fractions) or laparoscopic radical prostatectomy (eight laparoscopic, 42 robot-assisted) (NCT01584258). The study, at the primary endpoint of 2 years post-treatment, found that SABR patients reported significantly worse bowel subdomain scores (mean (SD) 88.7 (12.6) vs. 97.7 (5.3), *p* < 0.001), better EPIC sexual scores (59.3 (30.3) vs. 26.5 (19.2), *p* < 0.001), and better urinary incontinence scores (88.4 (18) vs. 73.4 (24.4), *p* = 0.03). Grade ≥ 2 GU toxicity was recorded in 5/53 (9.4%) SABR patients compared to 3/42 (7%) surgery patients (*p* = 0.97), with no grade ≥ 2 GI toxicity reported in either group [27].

### 2.5. The Role of ADT and SABR in Localised Prostate Cancer

The systematic review and meta-analysis performed by Jackson et al. reported that, although ADT was used in 654 (15%) of the 6116 patients analysed across 36 prospective studies, ADT use was not significantly associated (*p* = 0.91) with biochemical recurrence-free survival. A pooled analysis of the impact of ADT was not possible due to the lack of quantitative information, with further investigation required [2].

King et al. [28] performed a pooled analysis of 1100 patients with clinically localised prostate cancer across eight institutions enrolled in separate prospective phase II clinical trials from 2003 to 2011 and with a median follow-up time of 36 months. Patients received 26.25 Gy in four-to-five fractions of SABR, with 147 (14%) patients undergoing ADT in conjunction with SABR. This was delivered as a median 4-month course of neoadjuvant and concurrent ADT at the discretion of the treating team to low- (8%), intermediate- (15%), and high-risk (38%) patients. No difference in biochemical recurrence (PSA > 2 ng/mL above nadir) was observed with ADT (*p* = 0.71), even in the intermediate- and high-risk groups. This is postulated to be due to the high biological doses corresponding with ultrahypofractionation; however, the appropriateness of ADT use in patients undergoing prostate SABR remains an area for further study.

## 3. Metastatic Prostate Cancer

Metastatic prostate cancer remains incurable. Improving its management is an active research area, aiming to eradicate oligometastases, prolong treatment-free survival, and defer progression to castrate-resistant prostate cancer (CRPC) [29]. SABR appears to be a practical option for metastasis-directed therapy (MDT), given its convenient outpatient delivery, limited duration, and non-invasive nature. Studies, to date, have shown good local control with a low toxicity profile when used either alone or in combination with ADT in relation to oligorecurrent prostate cancer [30,31,32].

However, inconsistencies in terminology need to be harmonised to enhance the impact of SABR on metastatic prostate cancer. Rogowski et al. performed a systematic review of radiotherapy used in relation to oligometastatic prostate cancer. They observed, across 56 studies, that the term oligometastasis was inconsistently defined, with the cutoff varying between 3 and 5. Additionally, there was no consensus on the interval of time defining metachronous compared to occult synchronous diseases [33].

Another consideration is the increasing international adoption of PSMA-PET/CT during the staging of prostate cancer. Taking advantage of its growing availability, Ong et al. performed a small retrospective cohort study of 20 men to demonstrate the utility of PSMA-PET/CT in the identification of patients with true oligometastatic prostate cancer who would stand to gain the largest therapeutic benefit from SABR. The study demonstrates that PSMA-PET/CT-guided SABR was effective in managing oligometastatic prostate cancer, with significant PSA responses (60–70%) and high local progression-free survival rates (up to 93% after 12 months). Longer intervals between the initial curative treatment and SABR were associated with better distant progression-free survival and androgen deprivation therapy-free survival [34].

Siva et al. investigated the safety and efficacy of a single 20 Gy fraction of SABR for patients with one-to-three oligometastases in a prospective trial where 33 men (median age of 70 years) received SABR to the oligometastases, with a median follow-up of two years [35]. Secondary endpoints included local and distant progression-free survival, toxicity, QOL, and PSA response. A total of 20 patients had bone diseases, 12 patients had nodal diseases, and one patient had a mixed disease. SABR was able to be delivered as planned in 97% of cases. There was one grade 3 adverse event of a vertebral fracture. After 2 years, local progression-free survival was 93% (95% CI 84–100) and distant progression-free survival was 29% (95% CI 25–60). Over one third of the men did not progress and were free from ADT after 2 years. No significant difference in QOL was observed relative to baseline, supporting the feasibility and low morbidity associated with the use of a single fraction of SABR in this small sample size.

It is well known that hormone-sensitive prostate cancer (HSPC) and CRPC represent distinct disease states with respect to tumour biology and prognosis. It has been suggested that these be stratified into separate subgroups for the treatment of oligometastases using SABR [33]. Supporting this, Mercier et al. presented results from a single-centre retrospective study of 87 men with oligometastatic diseases (here defined as ≤3 active lesions) treated with SABR. A total of 19 patients had CRPC and 69 had HSPC at the time of SABR, with a median follow-up of 41.6 months. Greater benefits were reaped by the HSPC group; however, well-selected CRPC patients also appeared to benefit from treatment. The median biochemical progression-free survival was 11.7 months in both groups, and the median distant metastasis-free survival was 21.8 months (95% CI 16.9–43.2) in the HSPC group compared to 17.6 months (95% CI 6.7–26.2) in CRPC patients (*p* = 0.018). In total, 36 (41%) patients were free from clinical relapse after 18 months [36].

Given that the available treatments confer minimal survival advantage to CRPC patients, MDT via SABR is an intriguing area of research warranting further work. Deek et al. retrospectively evaluated 68 patients treated with SABR, with outcomes compared to oligoprogressive CRPC patients treated with systemic therapy alone. The median time to biochemical recurrence (9.7 vs. 4.2 months), the time to the next intervention (14.9 vs. 8.8 months), and the distant metastasis-free survival (12.7 vs. 8.9 months) were superior in patients treated with MDT, highlighting the merit of further prospective clinical trials evaluating this [37]. The updated 5-year results from the STOMP trial reinforced a significant difference in ADT-free survival favouring MDT (34%) compared to surveillance (8%), with an HR of 0.57 for surveillance (80% CI 0.38–0.84) [38].

### Immunomodulatory Effects of SABR

Although not as well studied as in the context of RCC, there has been interest in the immunomodulatory effects of SABR in relation to both localised and metastatic prostate cancer. Given the known low alpha–beta ratio and the high sensitivity of prostate cells to higher doses of radiation, further work optimising the combination of SABR and systemic therapy has been suggested [39]. In vitro, hypofractionation has been found to reverse the immunosuppressive tumour microenvironment, leading to increased CD8+ T cells and reduced myeloid-derived suppressor cells, an effect not seen with conventional fractionation [30]. The ORIOLE phase II randomised clinical trial, which investigated the effect of SABR on oligometastatic prostate cancer, detected significant tumour-specific T cell expansion post-SABR when sequencing the T cell receptor [29].

Metastatic prostate cancer has historically failed to show any significant response to immunotherapy, hypothetically due to its immunosuppressive microenvironment. The combination of SABR and immunotherapy holds great promise as a way to overcome this, especially in relation to metastatic CRPC given the limited treatment options and poor outcomes [31].

Zhang et al. reported the outcomes of a phase II trial evaluating SABR and T cell immunity in oligometastatic CRPC patients. A total of 89 men with oligometastatic CRPC were treated with SABR, and peripheral blood T cell subpopulations were analysed before and after SABR administration. Baseline high levels of tumour-reactive T cells predicted superior PSA, local, distant, and progression-free survival. Baseline high levels of effector memory T cells were associated with improved PSA progression-free survival. Notably, an increased number of tumour-reactive T cells 14 days post-SABR was associated with superior overall survival after 24 months. This was the first study to identify immunologic biomarkers to guide future randomised trials [32].

Additionally, the phase II OCEPAC clinical trial has shown that the combination of SABR with anti-PD1 immunotherapy can achieve very promising response rates in relation to heavily pre-treated metastatic CRPC [40]. This is concordant with the signal from the final analysis of the CA184–043 phase III randomised trial of ipilimumab versus placebo following conventional radiotherapy [41].

A summary of the utility of SABR in the treatment of prostate cancer can be found in Table 1.

## 4. SABR in Localised Renal Cell Carcinoma

RCC has traditionally been considered an intrinsically radioresistant tumour due to its poor response to normofractionation. Attempts to integrate conventionally fractionated radiation into RCC management over the past 50 years have shown limited success. While early retrospective data suggested survival benefits following pre-operative radiation, subsequent prospective trials have reported no significant survival advantage, such as van der Werf-Messing’s study (126 patients, 30 Gy in 15 fractions) and Juusela’s study (33 Gy in 2.2 Gy fractions, 5-year OS 47% vs. 63% for surgery alone) [42,43]. Post-operative radiotherapy has also failed to improve survival, as shown by the Copenhagen renal cancer study group (5-year survival 62% surgery alone vs. 38% surgery + 50 Gy radiotherapy) where it caused complications in 44% of patients [44]. As with prostate irradiation, advances in radiation techniques have allowed high doses to be delivered in a highly conformal and accurate manner, overcoming the radioresistance of RCC cells while minimising the dose to adjacent radiosensitive organs, particularly the GI tract [45].

When compared to prostate cancer cells, the radiobiology of RCC is less well delineated. The alpha–beta ratio of RCC is still unclear, with values between 2.6 and 6.92 suggested by Ning et al. [46]. As such, there is currently no consensus on the optimal SABR dose and fractionation for the primary management of RCC [45]. The sensitivity of RCC metastases to modern ultrahypofractionated doses has led multiple groups to investigate the utility of SABR for the primary treatment of RCC, with promising initial results [47].

RCC tumours are typically slow growing, showing a slow response to radiotherapy on conventional imaging with CT or MRI. Sun et al. conducted a retrospective cohort study of 41 tumours treated with SABR. The mean pre-treatment linear tumour growth rate was 0.68 cm annually compared to −0.37 cm following SABR, with persistent tumour enhancement after treatment. Furthermore, some tumours displayed an initial period of radiographic growth prior to shrinking. This highlights that accepted response criteria signifying local recurrence for thermal ablation, namely persistent radiographic enhancement and tumour diameter, are not applicable to SABR [48]. Ongoing work is currently evaluating PSMA-PET/CT as a means of assessing oncological responses in primary RCC [49].

Radiation may disrupt planes and compromise future surgical dissection, although most patients treated with SABR are poor operative candidates. Radiation-associated secondary malignancies have not been reported following SABR use for the treatment of localised RCC [45], but it remains a theoretical risk.

Per EAU guidelines, partial nephrectomy is the preferred management option in most patients, where technically feasible [50], for the treatment of T1 tumours. However, in elderly comorbid patients presenting with small renal masses, ablative techniques such as radiofrequency ablation (RFA) and thermal ablation are alternatives with higher local recurrence rates [51] which require higher baseline physiological fitness compared to SABR.

Correa et al. presented a systematic review and meta-analysis which evaluated SABR use in patients suffering from localised RCC, evaluating local control, toxicity, and renal function in 372 patients pooled from 26 studies, with a mean tumour size of 4.6 cm and a mean pre-SABR eGFR of 59 mL/min [47]. A total of 80% of the patients presented with localised RCC, with the remainder receiving SABR for metastatic diseases. In this combined patient cohort with a median follow-up of 28 months, local control was 97.2% (95% CI 0–4.3%), with a decrease in eGFR of 7.7 mL/min (range −12.5 to −2.8). A total of 1.5% of the patients experienced grade ≥ 3 toxicities (95% CI 0–4.3%), highlighting the importance of rigorous patient selection to minimise the toxicity risk to the adjacent organs, such as prior abdominal radiotherapy, inflammatory bowel disease, Von Hippel–Landau disease, or connective tissue disorders [45].

SABR offers adequate local control coupled with a low toxicity profile in those not fit for surgical management or thermal ablation [47,52]. The International Radiosurgery Oncology Consortium for Kidney (IROCK) performed a pooled multicentre analysis of 223 patients from nine institutions to investigate the use of single-fraction SABR in RCC patients (25 Gy in 118 patients) and the administration of 40 Gy in two-to-ten fractions [53]. At a median follow-up duration of 4 years, the local control rate was 97.8%, the cancer-specific survival was 91.9%, and the overall survival was 70.7%. Grade 1–2 adverse effects occurred in 35.6% of the patients, with only 1.3% experiencing grade 3–4 toxicity. A recent systematic review by Ali et al. has demonstrated that there is currently no data to guide the maximum safe dose to optimise local control while minimising toxicity [54].

### 4.1. Comparison with Thermal Ablation Techniques

There is currently a paucity of studies where the use of SABR is directly compared to the use of thermal ablation techniques, such as cryoablation, RFA, and microwave ablation [55]. Hence, these techniques will be discussed here in tandem.

Thermal ablation techniques are typically limited to small T1 renal masses and non-midpole tumours distant from the hilum and proximal ureter [50], as invasive local ablation may result in bleeding, strictures, or fistula formation [56]. In addition, independent of direct comparisons in terms of the safety and efficacy of SABR, uncertainties remain regarding the oncologic efficacy of thermal ablation. The EAU renal cell cancer guideline panel has concluded that the current paucity of studies limits evidence-based comparisons between thermal ablation techniques and the gold standard of partial nephrectomy in relation to localised renal masses [57].

Yet in general, thermal ablation results in local control rates of 83–95% compared to partial nephrectomy. SABR is well placed to be used in lieu of thermal ablation as a less invasive alternative suitable for more comorbid patients [58]. Additionally, SABR may also be more cost-effective [59].

However, head-to-head comparisons of tumour growth responses between thermal ablative techniques and SABR are challenging, as the established criteria for radiographic local recurrence in thermal ablation do not apply to SABR irradiated tumours [48]. The RADSTER trial, which has completed recruitment, may, in the future, clarify the suitability of SABR compared to RFA to overcome this lack of evidence-based guidance for patient selection (NCT03811665).

Another future consideration beyond short-term safety and efficacy is the suitability of post-SABR salvageability should future salvage, cytoreductive nephrectomy, or thermal ablation be required. A case report by Gorin et al. in 2015 describes the technical feasibility of salvage robot-assisted partial nephrectomy following robotic SABR [60]. Additionally, a pilot study by Singh et al. has shown that nephrectomy post neoadjuvant SABR is feasible and safe [61]. NAPSTER, a phase II randomised clinical trial, is currently investigating neoadjuvant pembrolizumab plus SABR prior to nephrectomy for RCC treatment [62].

### 4.2. Impact of SABR on Renal Function

The findings from IROCK include a small but significant fall in the median GFR (59.9 mL/min/1.73 m^3^ to 54.4 mL/min/1.73 m^3^) [63]. According to multiple studies, a decrease in eGFR of 5–15 mL/min is anticipated in the years after SABR, with the broad range due to heterogeneity in tumour size, location, and SABR regimens [47,64,65,66]. The 5-year outcomes from IROCK confirm these findings, with a median decrease in eGFR of 14.2 mL/min/1.73 m^3^ [63]. Compared to thermal ablation and partial nephrectomy, the fall in GFR following SABR is comparable; however, radical nephrectomy results in a more significant GFR decline, as would be anticipated [67]. Surprisingly, 52 patients (26.5%) had a higher GFR following SABR [63].

Nevertheless, candidates for SABR in the context of localised RCC are comorbid, with an expected decline in renal function further contributing to the effects of SABR [63]. A baseline eGFR > 30 mL is suggested by Siva et al. to reduce the risk of lifelong dialysis following SABR [52]. The group additionally suggests that T2b tumours are a reasonable size-based exclusion criteria when evaluating patient suitability for treatment with SABR. As reported by Glicksman et al., a larger tumour size corresponds to a greater loss in renal function due to a larger area of the functional parenchyma receiving ultrahyperfractionated dosing and to a lower sparing of the renal cortex [66]. Finally, given the paucity of studies on radiation for non-clear cell RCCs, it is unclear, at this point, if tumour characteristics impact the efficacy of SABR in treating other histologies [45].

### 4.3. Large RCCs

In IROCK, SABR use in patients with <T1b primary RCCs was effective, tolerable, with a modest decline in renal function [63]. Similarly, Correa et al. performed a retrospective analysis on 11 patients where SABR was safely and tolerably delivered to large renal tumours (median diameter of 9.5 cm), reporting five grade 1 toxicities and two grade 2–3 toxicities [67].

Based on these encouraging toxicity results, the group successively devised a prospective study investigating five-fraction SABR as an alternative to cytoreductive nephrectomy for large RCCs in 12 patients with metastatic RCC and a median tumour size of 8.7 cm [68]. After a median follow-up of 5.8 months, three grade 3 adverse events were recorded alongside a median tumour size reduction of −17.3% (range from +5.3% to −54.4%). In this relatively short follow-up, renal function was largely preserved, with no significant reduction in GFR observed 12 weeks following SABR administration; however, whether this preservation of renal function persists remains to be seen, as Glicksman et al. reported a larger decline in renal function in large renal masses at a median follow-up of 27.8 months [66].

### 4.4. SABR for IVC Tumour Thrombus

It is well established that thrombectomy is the only curative option in the presence of IVC tumour thrombus [50]. However, Freifeld et al. conducted a retrospective cohort study comprising 15 patients, of which 50% had level III or higher tumour thrombus. A dose of 72 Gy was delivered in one-to-five fractions, resulting in a median survival of 34 months and in a 58% response on imaging. No grade ≥ 3 adverse events were reported, leading the authors to suggest that SABR is feasible and safe, with symptom palliation reported in all patients in the cohort [69].

The use of neoadjuvant SABR (Neo-SABR) in the presence of IVC tumour thrombus is currently under investigation. Margulis et al. have published the initial safety results of an ongoing phase II trial inclusive of six RCC patients (three of whom presenting with metastatic diseases) with IVC tumour thrombus who have undergone Neo-SABR (40 Gy in five fractions), followed by radical nephrectomy and IVC thrombectomy [70]. Ninety days post-surgery, 78 grade ≤ 2 adverse events were reported. Three grade 3 events were reported, with no grade ≥ 4 events. After a median follow-up of 24 months, all patients survived, suggesting that Neo-SABR is feasible and safe. Of the three patients with metastases, one exhibited a complete response, while another exhibited a partial response without the concurrent use of systemic therapy. One patient developed a new metastatic disease. Further examination into the pathological and immunological changes attributable to SABR is ongoing.

Similarly, Liu et al. have published their protocol for an ongoing cohort trial investigating the safety and efficacy of Neo-SABR, with primary endpoints of adverse events 4–6 weeks post Neo-SABR, the level of tumour thrombus, recurrence-free survival, cancer-specific survival, and overall survival, reporting on intraoperative performance metrics and post-operative complications [71].

## 5. Metastatic RCC

The role of SABR in treating sites of metastases is more established than that in treating localised diseases. One third of the patients with RCC present with metastatic diseases, which occur in almost any soft tissues in the body, most commonly lung, bone, liver, and brain [61]. As SABR is non-invasive and suitable for frail patients, it has experienced increased use in relation to the management of oligometastatic RCC [72].

The spine is the most common site of bone metastasis. A randomised phase III trial performed by Sahgal et al. has shown that SABR is superior to conventional radiotherapy for palliative pain control in patients with a metastatic disease from several primary cancers, including RCCs [73]. Hussain et al. performed a retrospective study following the outcomes of 90 RCC patients with high-grade epidural cord compression treated with adjuvant SABR and separation surgery [74]. A total of 11% of the patients had solitary metastases, 33% presented with oligometastatic diseases, and 56% presented with widespread diseases. The median follow-up was 14.2 months for the cohort, and 38.3 months for survivors, achieving a local control rate of 95.4% (95% CI: 91.0–99.8%) one year post-surgery and a median overall survival of 14.8 months [74].

While SABR typically describes the delivery of highly conformal ablative doses of radiation to extracranial sites of diseases [45], SABR has also been applied to intracranial RCC metastases. A total of 10% of the patients with RCCs develop brain metastases resulting in significant morbidity and mortality, with a median overall survival of 6–12 months despite the advent of targeted therapies [75,76]. Promisingly, in a large retrospective analysis of 69 patients with a total of 146 RCC brain metastases, a local control rate of 96% and a median overall survival of 15 months were reported, with 83% of the patients instead died of extracranial disease progression [77].

Emerging evidence from several studies suggests that SABR may play a role in the management of oligometastatic diseases by delaying the need for systemic therapy and by maintaining QOL without sacrificing overall survival [54]. For instance, Tang et al. performed a phase II clinical trial whereby 30 patients with oligometastatic RCC (here defined as less than six lesions) were treated with SABR to evaluate its feasibility and efficacy in this setting [78]. The trial described a 1-year progression-free survival of 64%, with 82% of the patients being successful at deferring systemic therapy. Two grade 3 and one grade 4 adverse events were reported. Another phase II trial performed by Hannan et al. reported the outcomes of 23 patients with oligometastatic RCC (defined by less than three lesions) treated with SABR, with 91.3% of the patients free from systemic therapy after 1 year [79]. SABR has also shown promise in the treatment of oligoprogressive diseases in prospective phase II trials [80,81].

### Synergy with Immunotherapy

SABR has been postulated to simultaneously provide a cytoreductive and immunomodulatory effect [82]. Prolonged survival has been observed following SABR to metastatic lesions in patients suffering from oligometastatic RCC, owing to focal radiation resulting in distant immunologic effects in non-irradiated body sites. When this results in tumour responses in distant sites, this is termed an “abscopal effect” [83]. This has been postulated to be due to a synergistic immune-priming effect of SABR, proven in pre-clinical models [30,83,84].

Using a mouse model, Deng et al. has shown that the therapeutic blockade of the T cell programmed death ligand 1 (PD-L1) enhances T cell function and is upregulated in the tumour microenvironment after high-dose ionising radiation. In addition to local tumour regression, high-dose radiation and anti-PD-1 synergistically enhance T cell function. This alters the tumour microenvironment and provides in vitro evidence of the interaction between high-dose radiation and the PD-L1/PD1 axis, establishing the basis for the investigation of combination therapy, such as the PD-1 antibody pembrolizumab and radiotherapy [83].

Higher radiation doses recruit alternative cell death pathways, such as endothelial cell apoptosis via the ceramide pathway, important in highly vascularised tumours such as RCCs [85]. RCCs are known to be highly immunogenic, with radiation-induced immunogenic cell death pathways postulated to result in an abscopal effect on distant metastases in case reports [86,87].

Encouraging results were reported by the RAPPORT trial, a multicentre phase II trial investigating the safety and efficacy of SABR followed by a short course of pembrolizumab in patients suffering from oligometastatic clear cell RCC. Thirty patients with 83 oligometastases were treated, with a median follow-up of 28 months. The regime was very well tolerated, with four patients (13%) experiencing grade 3 adverse events and no grade ≥ 4 events recorded. Freedom from local progression was 92%, the objective response rate was 63%, and the disease control rate after 6 months was 83%. The estimated one- and two-year overall survival was 90% and 74%, respectively, and one- and two-year progression-free survival was 60% and 45%, respectively [88].

Further answers are on the horizon, with the SAMURAI trial (NCT05327686), a phase III randomised trial allocating patients to a group receiving SABR plus immunotherapy or to a group receiving immunotherapy alone. CYTOSHRINK (NCT04090710), a phase II randomised trial, is recruiting patients to investigate the efficacy of SABR administration followed by standard-of-care immunotherapy versus immunotherapy alone. Additionally, Neo-SABR is currently being investigated in patients suffering from localised RCC. The NAPSTER trial will investigate the role of Neo-SABR with or without the addition of pembrolizumab prior to nephrectomy [62].

A summary of the utility of SABR in RCC treatment can be found in Table 2.

## 6. Conclusions and Future Perspectives

SABR has emerged as a safe, effective, and feasible treatment for urologic cancers. SABR has the added benefit of being a suitable treatment for populations with significant comorbidities or surgical constraints. It has experienced an increased use in the treatment of localised prostate cancer and metastatic RCC, with good oncological outcomes and an acceptable toxicity profile. Additionally, the performance of SABR in metastatic prostate cancer and localised RCC treatment heralds promising early results in clinical trials conducted to date concerning toxicity and oncological outcomes.

Ongoing clinical trials are assessing the efficacy of SABR in innovative combinations with standard and investigational systemic treatments for prostate cancer. Arguably, there is sufficient evidence to support its adoption as a standard radiation therapy, especially in relation to localised disease treatment. Following SABR’s established use in metastatic RCC treatment, the established doctrine of its radioresistance to conventionally fractionated radiotherapy has been disproved with the advent of hypofractionation. Prospective and retrospective clinical trials have investigated the use of SABR in patients suffering from primary RCC, reporting high local control rates and acceptable toxicity.

Nevertheless, further larger-scale prospective clinical trials are underway to clearly define SABR’s niche in the multidisciplinary approach to the treatment of urologic cancers and subsequently update clinical practice guidelines. Of particular interest to urologists are two assessments comparing SABR to radical prostatectomy [29], with limited comparisons between SABR and partial nephrectomy in relation to localised disease treatment.

## Figures and Tables

**Table 1:** Utility of SABR in the treatment of prostate cancer.

Utility of SABR in the Treatment of Prostate Cancer	
Advancement	Details
Alpha–beta ratio	Low alpha–beta ratio (1.5 Gy) exploited for larger effective radiation doses [6]. Prostate cells survive small doses but are more likely to be destroyed with larger radiation doses [7].
Utility for localised disease	A systematic review and meta-analysis performed by Jackson et al. pooled 6116 men with localised prostate cancer from 36 prospective studies with a median follow-up of 39 months [2]. SABR resulted in excellent tumour control and improved biochemical control with increased doses, although it slightly worsened late GU toxicity.
Utility for metastatic disease	Good local control with a low toxicity profile, either alone or in combination with ADT in oligorecurrent prostate cancer patients [30,31,32]. Prolongs treatment-free survival and defers progression to CRPC [29,38].
Toxicity and QOL	Equivalent toxicity to conventional ERBT [11,12]. Toxicity appears to be favourable in comparison to brachytherapy [2]. Fewer radiation sessions compared to conventional radiotherapy reduce the burden of time spent receiving treatment on patients [5].
**Future Directions**	
Immunomodulatory effects	SABR in oligometastatic prostate cancer patients results in significant tumour-specific T cell expansion, predicting superior PSA, local, distant, and progression-free survival [29,32].
Comparison with surgery	Comparison with radical prostatectomy is ongoing (PACE-A) [27].

**Table 2:** Utility of SABR in RCC treatment.

Utility of SABR in RCC Treatment	
Advancement	Details
Alpha–beta ratio	Range of 2.6–6.92 [46]. Consensus lacking on optimal SABR dose and fractionation for primary RCC management [45,46]. RCC is traditionally considered radioresistant, but advances in radiation techniques allow high doses to overcome this while protecting radiosensitive organs [45].
Utility for localised disease	A systematic review and meta-analysis by Correa et al. evaluated SABR in patients suffering from localised RCC, finding acceptable local control and toxicity, with a minor decline in renal function in 372 patients pooled from 26 studies [47]. These findings are concordant with IROCK, which pooled 223 patients from nine institutions to investigate the efficacy of single-fraction SABR for RCC treatment [53].
Utility for metastatic disease	More established role compared to that in the treatment of localised diseases [72]. Superior to conventional radiotherapy with respect to palliative pain control in patients with metastatic diseases [73]. Delays systemic therapy and retains QOL without sacrificing the overall survival [54,78].
Toxicity and QOL	A decrease in eGFR of 5–15 mL/min is anticipated, depending on tumour size, location, and SABR regimens [47,64,65,66]. A baseline eGFR > 30 mL is suggested by Siva et al. to reduce the risk of lifelong dialysis following SABR administration [52]. Fewer radiation sessions compared to conventional radiotherapy are necessary to reduce the burden of time spent receiving treatment on patients [5].
**Future Directions**	
Immunomodulatory effects	The “abscopal effect”: SABR has been postulated to simultaneously provide a cytoreductive and immunomodulatory effect [82,83,84], with favourable freedom from local progression, disease control, and overall and progression free survival [88]. Several trials are underway (SAMURAI, CYTOSHRINK, NAPSTER).
Comparison with surgery	Limited comparisons to nephrectomy.

## Data Availability

Not applicable.
